# The roles of anti-Müllerian hormone in breast cancer

**DOI:** 10.1530/ERC-23-0060

**Published:** 2023-08-14

**Authors:** Xuan Chen, Sixuan Liu, Xue Peng, Xiangyun Zong

**Affiliations:** 1 Department of Breast Surgery, Shanghai Sixth People’s Hospital Affiliated to Shanghai Jiao Tong University School of Medicine, Shanghai, China

**Keywords:** anti-Müllerian hormone, hypothalamic–pituitary–ovarian axis, anti-Müllerian hormone receptor II, ovarian function, breast cancer

## Abstract

Anti-Müllerian hormone (AMH) is produced and secreted by granulosa cells of growing follicles, and its main role is to inhibit the recruitment of primordial follicles, reduce the sensitivity of follicles to follicle-stimulating hormone (FSH), and regulate FSH-dependent preantral follicle growth. It has become an effective indicator of ovarian reserve in clinical practice. Research on AMH and its receptors in recent years has led to a better understanding of its role in breast cancer. AMH specifically binds to anti-Müllerian hormone receptor II (AMHRII) to activate downstream pathways and regulate gene transcription. Since AMHRII is expressed in breast cancer cells and triggers apoptosis, AMH/AMHRII may play an important role in the occurrence, treatment, and prognosis of breast cancer, which needs further research. The AMH level is a potent predictor of ovarian function after chemotherapy in premenopausal breast cancer patients older than 35 years, either for ovarian function injury or ovarian function recovery. Moreover, AMHRII has the potential to be a new marker for the molecular typing of breast cancer and a new target for breast cancer treatment, which may be a link in the downstream pathway after TP53 mutation.

## Introduction

Anti-Müllerian hormone (AMH) is a homodimeric glycoprotein that belongs to the transforming growth factor-β family (Cate *et al.* 1986). The earliest role of AMH to be discovered was its role in male sexual differentiation (Jost 1947). It was later found that AMH also plays a role in the differentiation and development of the reproductive system in the female embryonic stage. It was initially called ‘hormone inhibitrice’ or ‘inhibiteur Müllerian,’ but today, it is better known as AMH or Müllerian-inhibiting substance (MIS) (Barbotin *et al.* 2019). The AMH gene was sequenced and cloned in mammals for the first time in 1986 (Picard *et al.* 1986). The development of sensitive tests in the early 1990s made it possible to measure AMH levels in serum, and the detection technology is still being improved (Dewailly *et al.* 2014). Current research on AMH has discovered its critical role in the hypothalamus–pituitary–gonad axis, thereby breaking the stereotype that it only functions in the ovary. In addition, studies found that anti-Müllerian hormone receptor II (AMHRII) is expressed in the breast during the occurrence of breast cancer, which is a hormone-dependent tumor. This implies that AMH may be closely associated with the occurrence, diagnosis, treatment, and prognosis of breast cancer. This review abstracts the complex relationships among AMH, the gonadal axis, and breast cancer, with the goal of providing novel ideas for the diagnosis and treatment of breast cancer.

## Constantly explore the new potential of AMH/AMHRII

### AMH: a follicular gatekeeper

At the beginning of the 20th century, some scientists focused their attention on AMH detected in females, which is mainly secreted by the granulosa cells of growing follicles, including preantral and small antral follicles, and it has some negative regulatory effects on the follicles. Data obtained from a large sample study indicated that AMH rises from birth and peaks at approximately 25 years old during puberty (Dewailly *et al.* 2014). It then maintains a steady decline until the levels are undetectable at an average age of 50–51 years (corresponding to menopause) (Dewailly *et al.* 2014). Several studies performed on various species have found that the number of growing follicles is significantly reduced after AMH is added to the ovary for cultivation (Durlinger *et al.* 1999, Durlinger *et al.* 2002, Gigli *et al.* 2005, Carlsson *et al.* 2006, Nilsson *et al.* 2007). In addition, primordial follicles are quickly depleted in the ovary after the AMH gene is knocked out (Reddy *et al.* 2010). Therefore, AMH has gradually been recognized as a negative regulator in the early stages of follicular development. To date, several effects of AMH on follicles have been discovered, including inhibiting the recruitment of primordial follicles (probably by downregulating several growth factors known to stimulate primordial follicle recruitment, such as Kit ligand and alkaline fibroblast growth factor) (Shahrokhi *et al.* 2018), regulating follicle growth during the gonadotropin-responsive phase (Kereilwe *et al.* 2018), reducing the sensitivity of follicles to follicle-stimulating hormone (FSH), thus limiting FSH-dependent preantral follicle growth (Durlinger *et al.* 2001, Dewailly *et al.* 2014), and restraining the activity of aromatase, thereby leading to a decrease in estrogen biosynthesis, which in turn affects follicle growth (Garrel *et al.* 2019), interfering with the meiosis of oocytes (Bedenk *et al.* 2020), preventing granulosa cell proliferation (Dewailly *et al.* 2016), and controlling follicular atresia (Kereilwe *et al.* 2018). It is worth noting that the concentration of AMH in antral follicles decreases as the diameter of the follicle increases, and the decrease is attributed to the choice of dominant follicles during the menstrual cycle (Dewailly *et al.* 2014, Dewailly & Laven 2019, Bedenk *et al.* 2020).

### AMH: a biomarker of ovarian reserve

Ovarian reserve refers to the number of primordial follicles in the ovarian cortex, thus indicating the ability of the ovary to produce fertilizable eggs. Currently, ultrasound-guided antral follicle counting (AFC) is the diagnostic procedure used to evaluate ovarian reserve. Some scholars have suggested that the level of serum AMH can indirectly reflect the size of the primordial follicle pool in the ovary or the abundance of ovarian reserve (Wong & Anderson 2018, Moolhuijsen & Visser 2020). Several studies have confirmed that the serum AMH level is positively correlated with AFC (Fanchin *et al.* 2003, La Marca *et al.* 2010, Lambertini *et al.* 2018, Silva *et al.* 2019). AMH levels can reflect ovarian function for women at a given age more sensitively than other reproductive hormones because the level of AMH starts declining earlier than other ovarian signs with the corresponding age (Shahrokhi *et al.* 2018). The dominant follicles and corpus luteum do not secrete AMH because AMH does not depend on the hypothalamic–pituitary–ovarian axis (Visser *et al.* 2012), thus making it stable during the menstrual cycle (Tsepelidis *et al.* 2007, Dewailly *et al.* 2014, Lambert-Messerlian *et al.* 2016). With regard to sensitivity and specificity, AMH is more suitable than FSH, estradiol, and inhibin B for the evaluation of ovarian reserve (Fanchin *et al.* 2003). Regarding AMH as an outstanding candidate to evaluate adult female ovarian function has become an epidemic (Dewailly & Laven 2019). The obtained results are then applied for individualized counseling of women with fertility needs to predict the menopausal date and reproductive life, to predict ovarian response to overstimulation in assisted reproductive technology, to assist in the diagnosis of ovarian diseases (such as polycystic ovary syndrome and congenital ovarian insufficiency), to evaluate the iatrogenic injury caused by chemotherapy, radiotherapy, and ovarian surgery, and finally to forecast ovarian function recovery. All these studies have obtained valuable results, but AMH as a marker of ovarian reserve still has some limitations, one of which is that it varies widely among individuals and it is difficult to set a reference value (Dewailly *et al.* 2014).

### AMH/AMHRII signaling pathways

There are two types of AMH receptors: type I and type II. AMHRII (also known as MIS receptor II) acts as a transmembrane sensor with serine/threonine protein kinase activity (Rak *et al.* 2019). It has been demonstrated that free AMH exclusively binds to AMHRII (Mishina *et al.* 1996, Josso *et al.* 2001), and the receptor regulates gene transcription via three mechanisms (Rak *et al.* 2019). First, when AMH binds to AMHRII, it forms a receptor complex with AMHRI. This complex phosphorylates AMHRI and activates serine/threonine protein kinase. Subsequently, intracellular Smad-proteins (R-SMads1/5/8) detach from the receptor complexes, allowing several proteins to enter the nucleus to regulate gene expression (Josso *et al.* 2001, Bedenk *et al.* 2020). In the nucleus, Smad proteins can either bind to DNA at Smad recognition sites (leading to transcriptional activation) or form complexes with other transcription cofactors that specifically affect the expression of target genes (Pankhurst *et al.* 2016, Rak *et al.* 2019). Second, binding of AMH to the type II receptor increases the content of free β-catenin in the cytoplasm, which then enters the nucleus together with lymphoid enhancer factor 1, upregulating the transcription of target genes (Rak *et al.* 2019). Third, the combination of AMH and AMHRII triggers the dissociation of heteromeric complexes of the nuclear factor kappa-B (NF-κB) family transcription factors (P50, P65, P52, and c-rel) and inhibitory proteins IκBα, which actuate the free transcription factors to enter the nucleus and trigger the transcription of the IER3 gene (Rak *et al.* 2019). IER3 encodes the IEX-1S factor, which is an early response protein to radiation exposure or γ-interferon or tumor necrosis factor-α (Segev *et al.* 2002). The third pathway is revealed to function in the mammary gland (Segev *et al.* 2000, Rak *et al.* 2019).

### AMHRII is highly expressed in the gonad axis and extragonadal organs

Interestingly, AMHRII traces have been found in several extragonadal organs, such as lung, pituitary, hypothalamus, and motor nerves (Garrel *et al.* 2016, Barbotin *et al.* 2019, Barret *et al.* 2021, Silva & Giacobini 2021), indicating that AMHRII is not only expressed in cells of gonad-related organs, including testicular Sertoli and interstitial cells, ovarian membrane and granulosa cells, prostate, endometrium, and breast ductal epithelium (Segev *et al.* 2001, 2002, Wang *et al.* 2009, Pfennig *et al.* 2015). This suggests that AMH may have more roles in addition to the already elucidated reproductive functions. Additionally, AMHRII has also been found to be expressed in multiple cancer cell lines, such as cervical cancer, endometrial cancer, ovarian epithelial cancer, and breast cancer (Peluso *et al.* 2014, Vaz-Luis & Partridge 2018). Therefore, AMH can unexpectedly contribute to the reduction of the growth and metastasis of breast cancer cells (Segev *et al.* 2000, Chang *et al.* 2011). All these discoveries have cast a mysterious veil on the true face of AMH and its receptors. Future research might also uncover more responsibilities of AMH and its receptors in the process of other diseases and tumors.

### AMH/AMHRII participates in the hypothalamic–pituitary–ovarian axis

**Figure 1:**
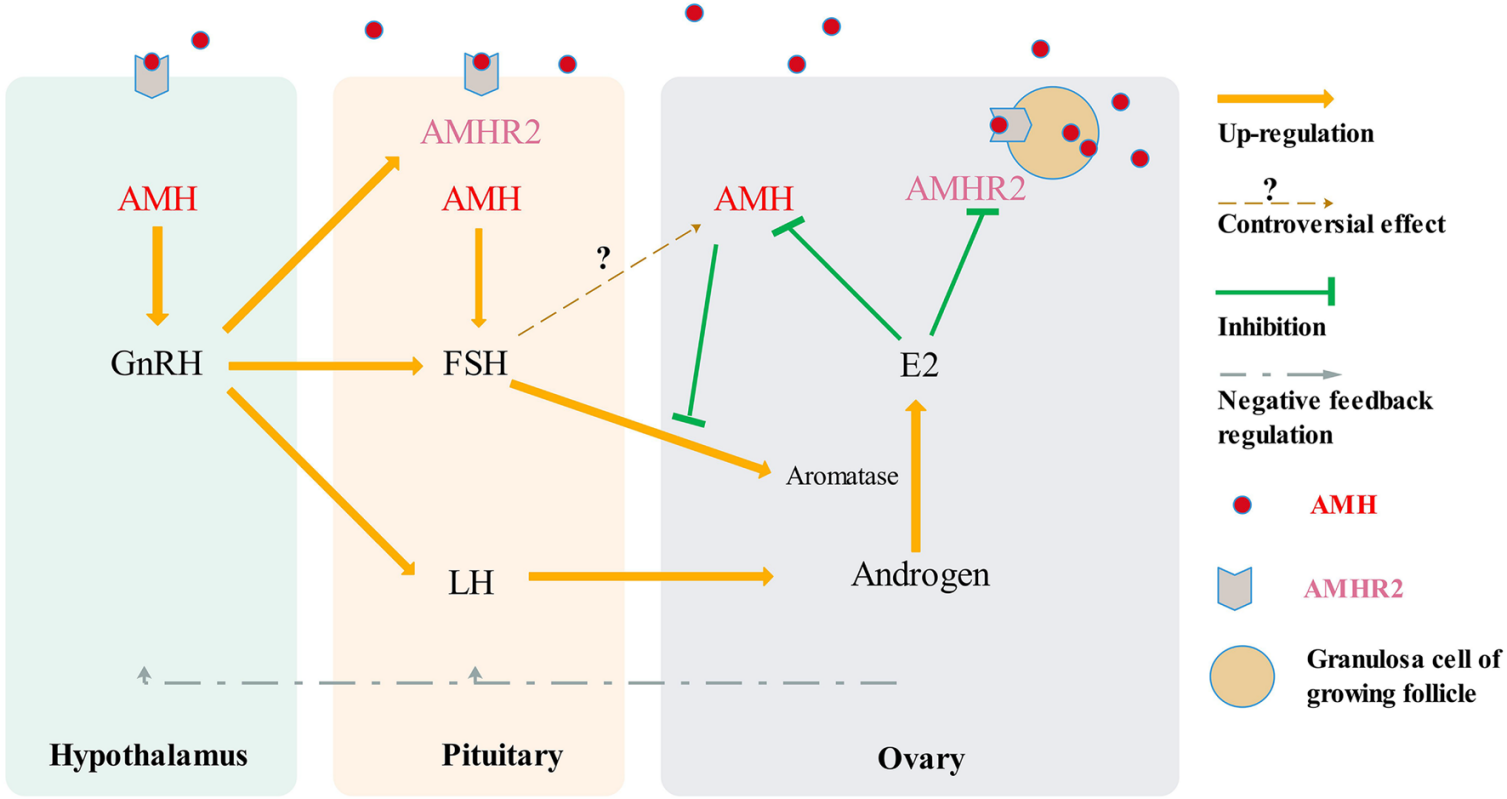
AMH/AMHRII participates in the hypothalamic–pituitary–ovarian axis. AMH secreted from granulosa cells of growing follicles can target AMHRII, which is located in the hypothalamus, pituitary, and ovary, interacts with hormones secreted from these organs, and forms a complex hormonal network. A full colour version of this figure can be found at https://doi.org/10.1530/ERC-23-0060.

Several studies have reported that mature neurons in adult brains exhibit high levels of AMH receptors (Wang *et al.* 2005, Barbotin *et al.* 2019, Silva & Giacobini 2021). This indicates that the focus of scientists for AMH is not only concentrated on the ovaries, with many studies focusing on the role of AMH in neuroendocrine both in physiological and pathological conditions. According to existing studies, the AMH signaling pathway is involved in the production and secretion of gonadotropin-releasing hormone (GnRH) (Ciofi *et al.* 2009, Schaeffer *et al.* 2013, Cimino *et al.* 2016, Prevot *et al.* 2018, Barbotin *et al.* 2019, Malone *et al.* 2019), plays a critical role in the differential regulation of gonadotropin (Durlinger *et al.* 2001, Bédécarrats *et al.* 2003, Garrel *et al.* 2016, Kereilwe *et al.* 2018, Garrel *et al.* 2019), and interacts with reproductive hormones in ovaries (Baarends *et al.* 1995, Bao *et al.* 1997, Evans & Fortune 1997, Durlinger *et al.* 2001, Ciofi *et al.* 2009, Deroo & Buensuceso 2010, Grynberg *et al.* 2012, Pierre *et al.* 2013, Dewailly *et al.* 2014, 2016, Kereilwe *et al.* 2018, Devillers *et al.* 2019, Umer *et al.* 2019) (Fig. 1). But these questions are not the focus of this review.

## AMH in breast cancer

### AMH may be associated with the occurrence of breast cancer

Breast cancer, as we know, is a kind of hormone-dependent malignancy. Because AMH has extensive interaction with hypothalamic–pituitary–gonadal axis and its corresponding reproductive hormones, including gonadotropin-releasing hormone, luteinizing hormone (LH), estrogen, progesterone, etc. (Baarends *et al.* 1995, Bao *et al.* 1997, Evans & Fortune 1997, Durlinger *et al.* 2001, Bédécarrats *et al.* 2003, Ciofi *et al.* 2009, Deroo & Buensuceso 2010, Grynberg *et al.* 2012, Schaeffer *et al.* 2013, Pierre *et al.* 2013, Cimino *et al.* 2016, Kereilwe *et al.* 2018, Prevot *et al.* 2018, Devillers *et al.* 2019, Garrel *et al.* 2019, Malone *et al.* 2019, Umer *et al.* 2019), AMH may have certain influence on the occurrence of breast cancer. Two small cross-sectional studies have been conducted: one reported significantly lower AMH levels in 22 women diagnosed with cancer or precancerous lesions compared with 8 women with benign biopsies (McCoy *et al.* 2011), and the other one reported no significant difference in AMH levels between breast cancer cases and healthy controls (Su *et al.* 2013). The results obtained from three prospective epidemiological studies have indicated a significant positive association between premenopausal plasma AMH levels and the risk of breast cancer (Dorgan *et al.* 2009, Nichols *et al.* 2015, Eliassen *et al.* 2016). Joanne *et al.* reported that there was an association between AMH and breast cancer, but they included more patients with a history of breast cancer in a first-degree relative in the case group than in the control group (18.1% vs 7.4%; *P* = 0.007) (Dorgan *et al.* 2009). Moreover, there was a closer connection between AMH and breast cancer for women who were diagnosed with breast cancer at an older age (more than 45 years old) than for those who were diagnosed at a younger age (less than 45 years old) (Dorgan *et al.* 2009). This may be attributed to the higher AMH levels, which delay the start of menopause, thus increasing the exposure to endogenous estrogen (Dorgan *et al.* 2009). In a large consortium study, Ge *et al.* confirmed that AMH is associated with breast cancer risk, with a 60% increase in risk for women in the top vs bottom quartile of AMH (Ge *et al.* 2018). But this conclusion has been strongly disputed by Blumenfeld, who believes that the association of high AMH and breast cancer may be due to a higher prevalence of PCOS in the high AMH group and not due to high AMH, per se (Blumenfeld 2019). However, this is also a controversial topic, because most of the literature has shown no significant association between PCOS and breast cancer risk (Carvalho *et al.* 2019, Meczekalski *et al.* 2020), and only a few studies have shown that PCOS may be associated with ER+ breast cancer (Wu *et al.* 2020).

However, the results from prospective epidemiological studies are inconsistent with laboratory results, which indicate an inhibitory effect of AMH in breast carcinogenesis. Scientists have attributed this inconsistency to the fact that the level of AMH treatment in the laboratory is significantly higher than human physiological levels. We believe that AMH levels in humans are not sufficient to resist the effects of estrogen on breast cancer cells. In addition, AMH levels in prospective epidemiological studies were not obtained at the time of breast cancer diagnosis. Consequently, it is worth investigating whether AMH could be a predictor for the occurrence of breast cancer.

### AMH/AMHRII inhibits the growth of breast cancer

**Figure 2:**
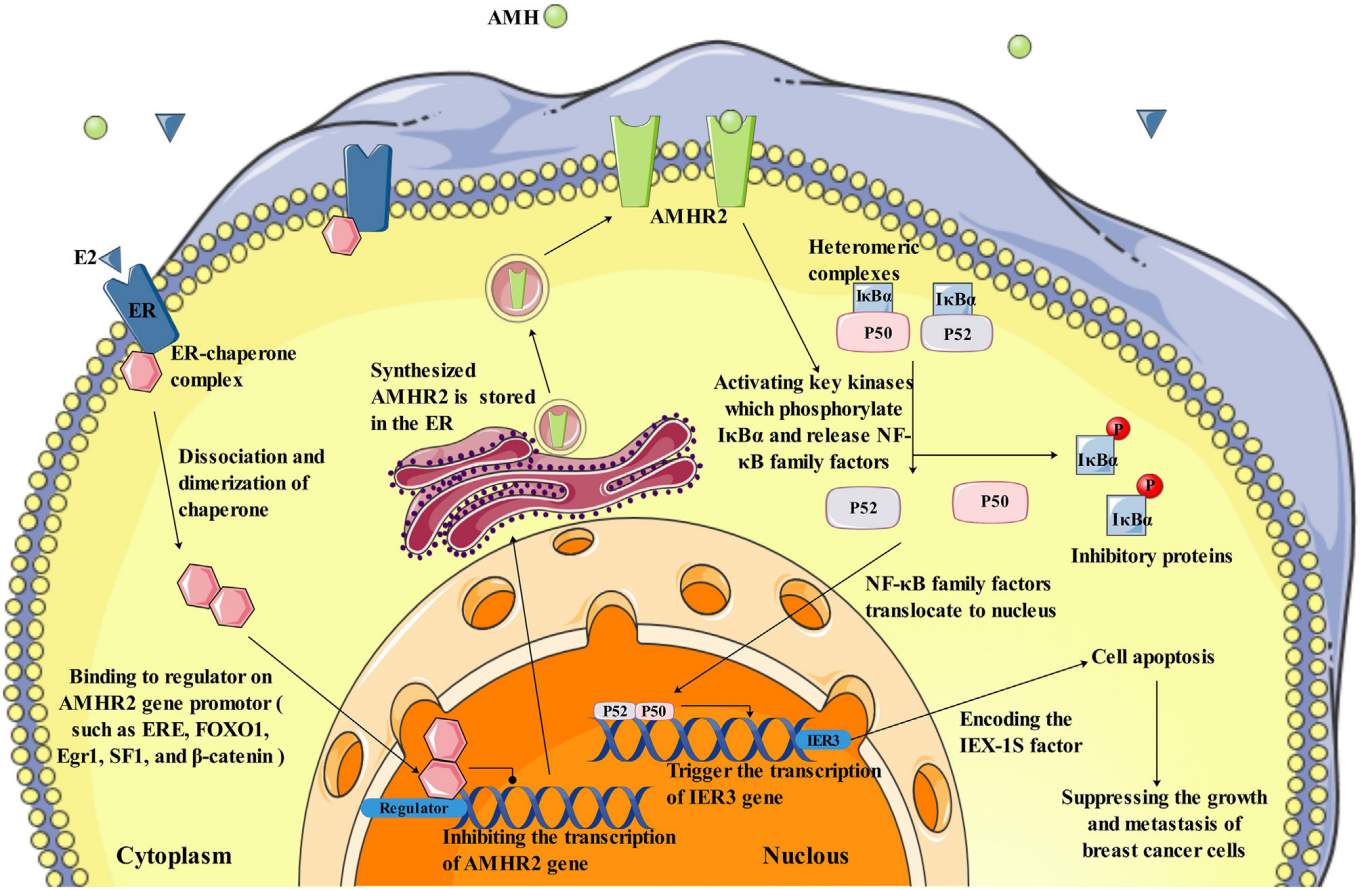
Possible mechanisms of AMH/AMHR2 in breast cancer. The hypothesis that estrogen may inhibit the expression of AMHRII in ER+ breast cancer cells and the mechanism by which AMH targets AMHRII leads to apoptosis in breast cancer was shown in the figure. A full colour version of this figure can be found at https://doi.org/10.1530/ERC-23-0060.

Several studies have reported that estrogen can inhibit the mRNA expression levels of AMH and AMHRII in the ovary (Grynberg *et al.* 2012, Pierre *et al.* 2017) and induce the expression of PR in the mammary gland (Lanari *et al.* 2009). AMHRII is also expressed in malignant breast tumors (Segev *et al.* 2000). So we hypothesize that estrogen may inhibit the expression of AMHRII in ER+ breast cancer cells. Therefore, the higher the abundance of ER on the surface of breast cancer cells, the more estrogen binds to ER. This binding may have an effect on regulators of the AMHRII gene promoter (ERE, FOXO1, Egr1, SF1, and β-catenin), may inhibit AMHRII transcription and expression, and may lower the abundance of AMHRII on the surface of breast cancer cells. The binding of AMH and AMHRII on the surface of breast cancer cells is also attenuated, thus blocking an apoptotic pathway of breast cancer cells to some extent. Reducing the level of estrogen or suppressing ER to upregulate the level of AMHRII on the surface of breast cancer cells or increasing the concentration of circulating AMH may enhance AMH binding to AMHRII on a large scale. Their binding can release NF-κB family transcription factors by phosphorylating the inhibitory protein IκBα and transferring free transcription factors into the nucleus, thereby selectively upregulating the early gene IEX-1S, which induces apoptosis of breast cancer cells. This can be considered a new idea for the treatment of breast cancer in the future (Fig. 2).

**Figure 3:**
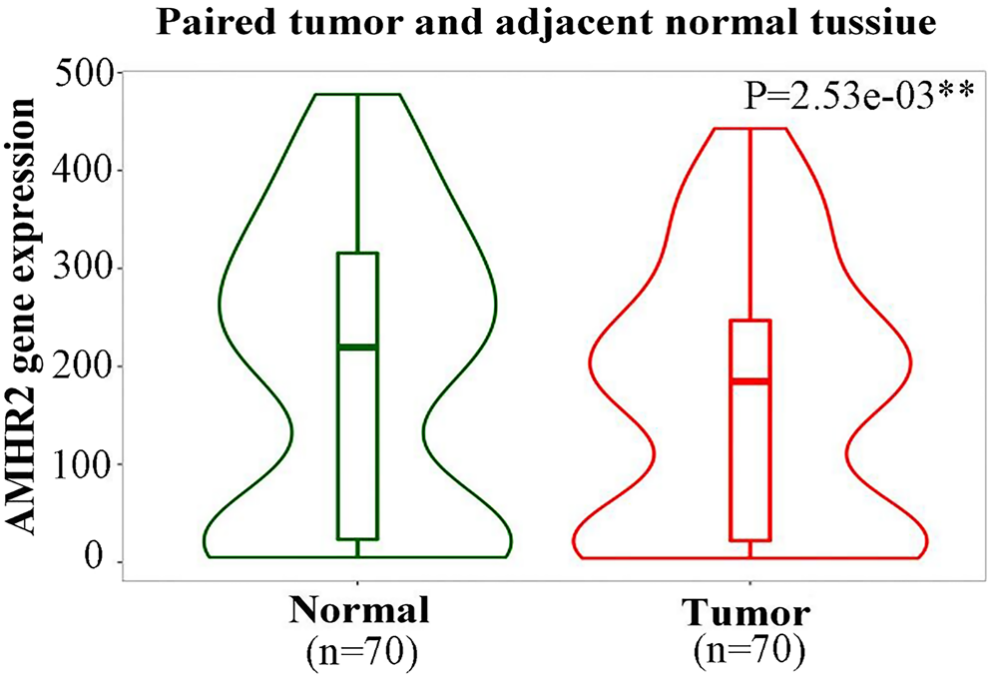
AMHR2 gene expression is negatively correlated with breast cancer. A violin plot comparing AMHR2 expression in breast tumor samples (*n* = 70) and adjacent normal tissue (*n* = 70). *P < 0.05, **P < 0.01, and ***P < 0.001. A full colour version of this figure can be found at https://doi.org/10.1530/ERC-23-0060.

**Figure 4:**
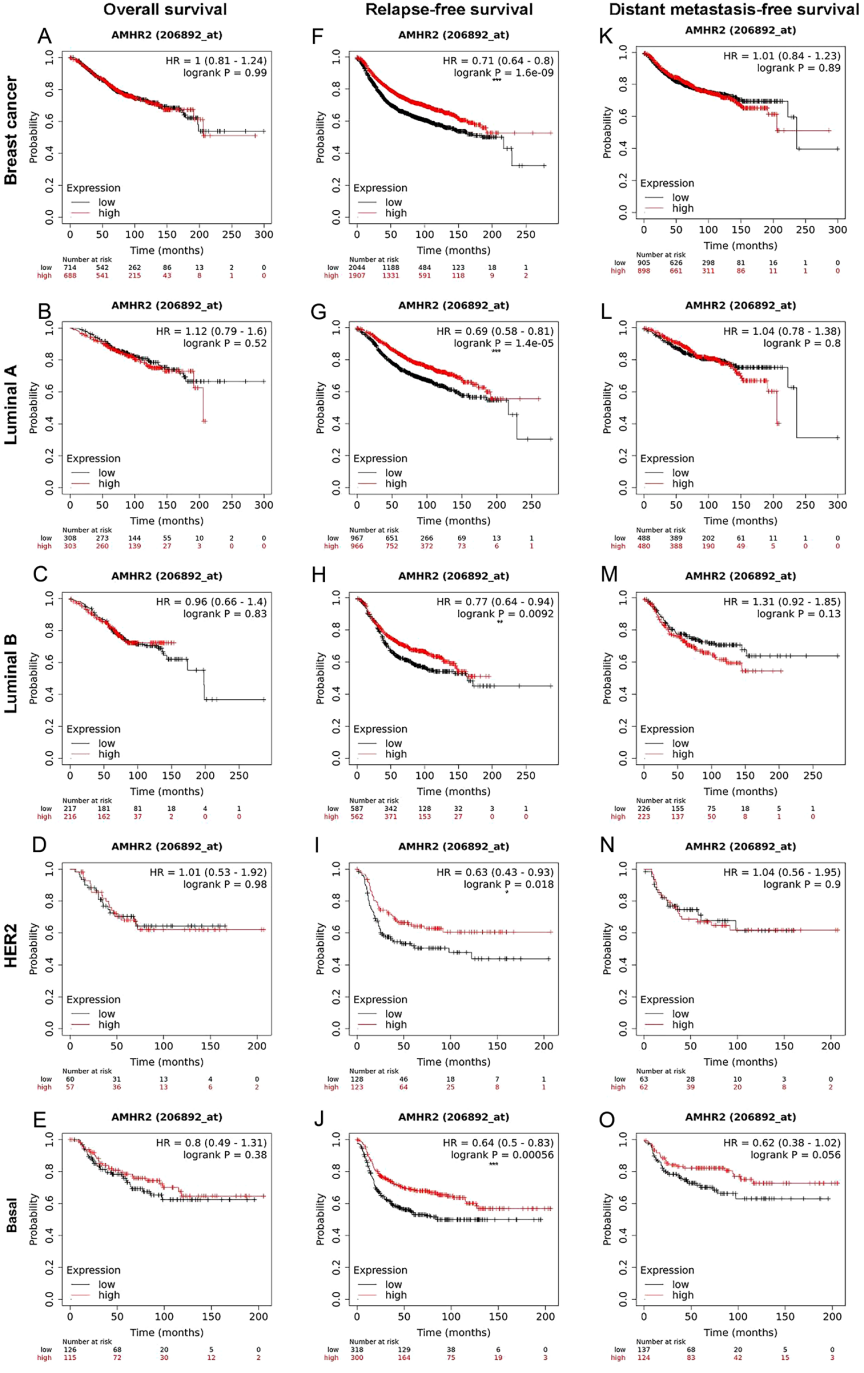
The level of AMHR2 gene expression in breast cancer tumors can predict the prognosis. (A–E) Overall survival according to the AMHR2 gene expression. (F–J) Relapse-free survival according to the AMHR2 gene expression. (K–O) Distant metastasis-free survival according to the AMHR2 gene expression. (A), (F), and (K) Overall breast cancer. (B), (G), and (L) Luminal A breast cancer. (C), (H), and (M) Luminal B breast cancer. (D), (I), and (N) HER2 breast cancer. (E), (J), and (O) Basal like breast cancer. *P < 0.05, **P < 0.01, and ***P < 0.001. A full colour version of this figure can be found at https://doi.org/10.1530/ERC-23-0060.

Many scientists have shifted their focus to laboratory studies with the goal of determining the relationship between AMH and breast cancer. The results obtained after *in vitro* experiments indicated that AMH can trigger cell apoptosis by releasing NF-κB family proteins and selectively upregulating the early gene IEX-1S. It can also inhibit the growth of normal mammary MCF10 cells (Segev *et al.* 2001) and both ER+ and ER− breast cancer cell lines (Segev *et al.* 2000). *In vivo* AMH administration was associated with a reduction in palpable breast tumors and enhanced apoptosis of ductal epithelial cells in mice (Segev *et al.* 2001, Gupta *et al.* 2005). Our team applied the online data analytic tool TNMplot (https://www.tnmplot.com/) and Kaplan–Meier Plotter (https://www.kmplot.com) from the GEO and TCGA databases and found that the expression of the AMHR2 gene in breast cancer tissues was lower than that in adjacent tissues and that those with higher AMHR2 gene expression had higher relapse-free survival (Fig. 3 and 4). The data from laboratories and databases indicate an inhibitory effect of AMH/AMHRII in breast carcinogenesis.

### Serum AMH can predict post-chemotherapy ovarian function in premenopausal women

As an important systemic treatment of cancer, chemotherapy is an effective way to reduce recurrence and improve survival rate. In addition to their anti-cancer effects, chemotherapeutic agents also have toxic effects on normal tissues and cells that are active in metabolism or proliferation. For instance, toxic effects on ovaries can lead to premature ovarian insufficiency (POI), ovarian failure, and sterility (Bedoschi *et al.* 2016). The mechanisms by which chemotherapeutic drugs damage the ovarian reserve are mainly divided into direct and indirect aspects. Chemotherapeutic drugs can cause direct toxicity to all levels of follicles, granulosa cells, membrane cells, blood vessels, and other ovarian stroma. Studies have also shown that excessive recruitment of primordial follicles causes indirect exhaustion of primordial follicles (Cui *et al.* 2018, Sonigo *et al.* 2019).

Of all chemotherapeutic drugs, alkylating agents are the most toxic to ovaries, among which cyclophosphamide (CTX) is widely used in the treatment of a variety of malignant tumors and autoimmune diseases, and it is also the basic ingredient of first-line chemotherapy for breast cancer (Jiang *et al.* 2013). CTX can directly damage the DNA of all levels of follicles (Barret *et al.* 2021). It has also been noted that granulosa cells of growing follicles, which can produce and secrete AMH, are particularly sensitive to alkylating agents (Yeh *et al.* 2009, Horicks *et al.* 2015, Lambertini *et al.* 2019). The results from a clinical study that we conducted indicated that the AMH levels of premenopausal breast cancer patients over 35 years old were reduced after CTX-based chemotherapy, and the level of serum AMH before chemotherapy can be used as a predictor of menstrual recovery after chemotherapy (Li *et al.* 2020). However, there was no significant change in the level of AMH after CTX-based chemotherapy for patients younger than 35 years (Li *et al.* 2020, Yu & Zong 2022). This can be attributed to the abundance of primordial follicular reserves in younger women, indicating that primordial follicles can be activated into growing follicles to maintain stable AMH levels after the damage and depletion of growth follicles by toxic chemotherapy drugs. These results are consistent with other clinical studies suggesting that AMH can be used as a predictor of ovarian reserve in older premenopausal patients after chemotherapy (Anderson *et al.* 2017, Wong & Anderson 2018). Additionally, we found that patients with lower baseline AMH levels are more likely to suffer from POI after chemotherapy (Zong *et al.* 2022*a*,*b*). However, some researchers have suggested that despite AMH correctly predicting the number of follicles or oocytes after any treatment, it cannot predict the amount of genetic damage that ultimately determines embryo quality and subsequent pregnancy (Dewailly & Laven 2019).

### AMH reverses ovarian injury caused by chemotherapy

**Figure 5:**
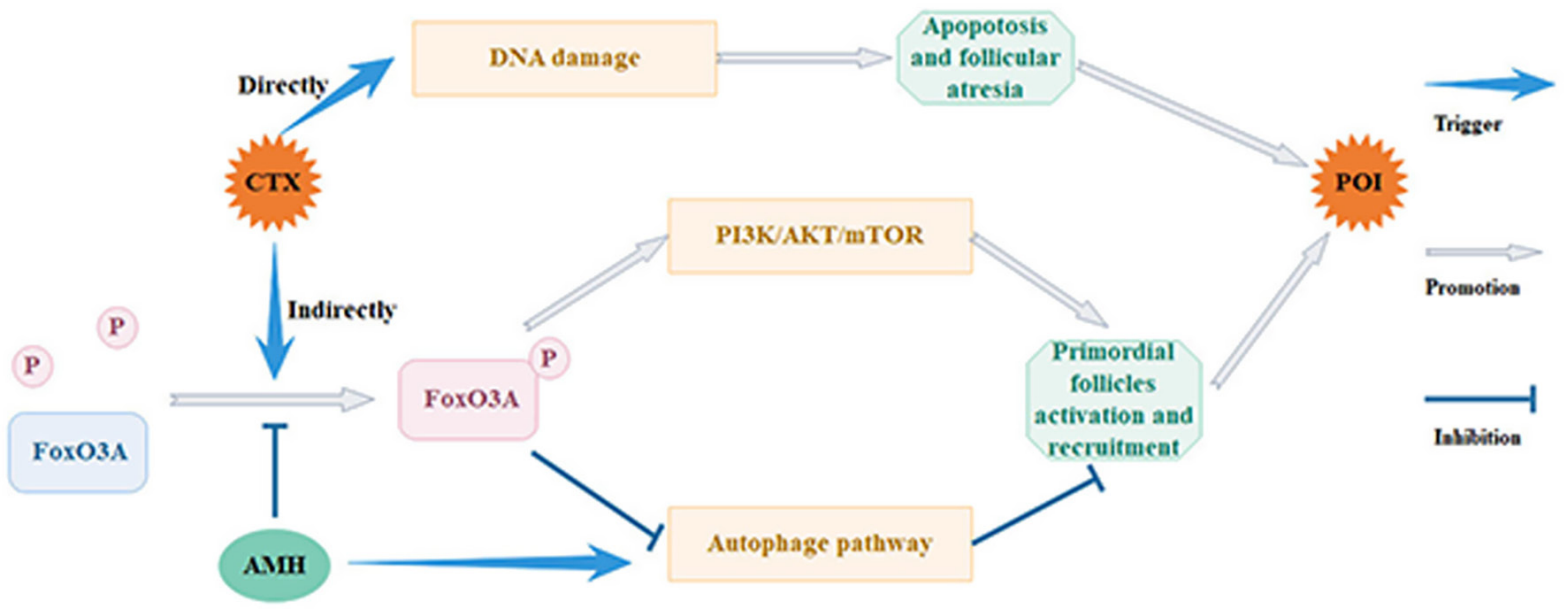
The mechanism by which AMH protects ovarian function during CTX. AMH can inhibit the activation of primordial follicles by preventing CTX-induced phosphorylation of FoxO3A, which can activate the PI3K/AKT/mTOR pathway and suppress autophagy to consume primordial follicles. Meanwhile, AMH can directly trigger autophagy to inhibit the activation and recruitment of primordial follicles and prevent POI. A full colour version of this figure can be found at https://doi.org/10.1530/ERC-23-0060.

A study in mice reported that injecting super physiological doses of recombinant AMH can limit the loss of primordial follicles caused by CTX, doxorubicin, or cisplatin (Kano *et al.* 2017). Current studies have found that AMH reverses CTX-induced loss of primordial follicles and suppression of ovulation (Sonigo *et al.* 2019). There is a common belief in the scientific community that the PI3K/AKT/mTOR signaling pathway is one of the mechanisms that regulates the resting state and survival of primordial follicles (Reddy *et al.* 2010). FoxO3A is a vital molecule in this pathway since it can activate primordial follicles when it is phosphorylated (Adhikari & Liu 2009). A study reported that treating the ovaries of mice with AMH can inhibit the recruitment of primordial follicles by preventing CTX-induced phosphorylation of FoxO3A (Cui *et al.* 2018). The study also found that AMH can play a role in preserving follicular pool reserves by promoting autophagy because FoxO3A is also a regulatory molecule in the autophagy pathway. These results are consistent with previous studies that reported that autophagy is involved in ovary reserve regulation (Sun *et al.* 2018, Delcour *et al.* 2019). Therefore, AMH protects ovarian function during chemotherapy by inhibiting the phosphorylation of FoxO3A, which plays a further role in the activation of primordial follicles (Fig. 5).

We acknowledge that cancer is formed by multiple genetic abnormalities and can be resistant to chemotherapy drugs. Therefore, therapies that simultaneously inhibit multiple signaling pathways involved in tumor cell proliferation may be more effective. From this viewpoint, a combination of AMH and cytotoxic agents may be ideal for AMHRII-specific antineoplastic therapy. In some AMHRII-positive human ovarian malignant tumor cell lines, the application of AMH in combination with cytotoxic drugs (adriamycin, platinum, paclitaxel, and rapamycin) provides synergistic effects or additive effects. Whether this combination therapy is beneficial against breast cancer requires further investigation.

### AMH may strengthen endocrine therapy for breast cancer

The positive expression of hormone receptor (HR+) is an objective indication for endocrine therapy regardless of the metastatic state of the breast cancer and the expression state of human epidermal growth factor 2 (HER2). Currently, there are three types of endocrine drugs that are administered. The first type is estrogen receptor modulators, including selective estrogen receptor antagonists and selective estrogen receptor downregulators, which can competitively inhibit the binding of estrogen and estrogen receptors. The second type is aromatase inhibitors, which reduce circulating estrogen levels by reducing the conversion of androgens to estrogens. The third type is gonadotropin-releasing hormone analogues (GnRHa), which can suppress ovarian function by inhibiting the secretion of LH and FSH (Li *et al.* 2019). Accumulating evidence indicates that endocrine therapy can reduce the effect of estrogen on micrometastatic tumor cells (MacLaughlin & Donahoe 2010). Therefore, it can significantly benefit HR+ breast cancer patients and reduce the 5-year recurrence rate (Waks & Winer 2019). With regard to our hypothesis, the reduction in circulating estrogen levels caused by endocrine therapy may decrease the inhibition of AMHRII expression on the surface of breast cancer cells by estrogen; thus, more AMH can bind to its receptor and induce tumor cell apoptosis. This explains the effect of endocrine therapy on anti-breast cancer cells from the perspective of AMH.

In recent years, GnRHa, as a kind of adjuvant endocrine therapy, has been applied in the treatment of premenopausal HR+ breast cancer patients (Cuzick *et al.* 2007, Yang *et al.* 2013, Del Mastro *et al.* 2016, Pagani *et al.* 2016). Most of the clinical guidelines or consensus for cancer management recommend the use of GnRHa in younger premenopausal breast cancer (Jackisch *et al.* 2015, Burstein *et al.* 2016, Paluch-Shimon *et al.* 2020). Studies have confirmed that GnRHa can reduce the secretion of FSH from the pituitary, causing ovarian function suppression (OFS) (Roness *et al.* 2014). We found that the serum AMH level in the CTX + GnRHa group was higher than that in the CTX group in mice or breast cancer patients (Li *et al.* 2020, 2021, Zong *et al.* 2022*b*). *In vitro*, CTX alone has been found to induce endoplasmic reticulum stress (ERS) in human ovarian granulosa cells, which decreased the release of AMH from cells (Li *et al.* 2021). The opposite effects were obtained in the CTX + GnRHa group. The secretion of AMH from granulosa cells was enhanced through GnRHa suppressing the mTOR pathway and activating autophagy, which relieved ERS induced by CTX (Li *et al.* 2021). Given that the high-frequency pulses of GnRH have been shown to upregulate AMHRII in the pituitary LβT2, studies should explore whether GnRHa can upregulate AMHRII expression on the ovary to protect ovarian reserve during chemotherapy or whether GnRHa can upregulate AMHRII expression on the surface of breast cancer cells, thereby increasing apoptosis of breast cancer cells and delaying disease progression.

### AMH and breast cancer therapeutic response

**Figure 6:**
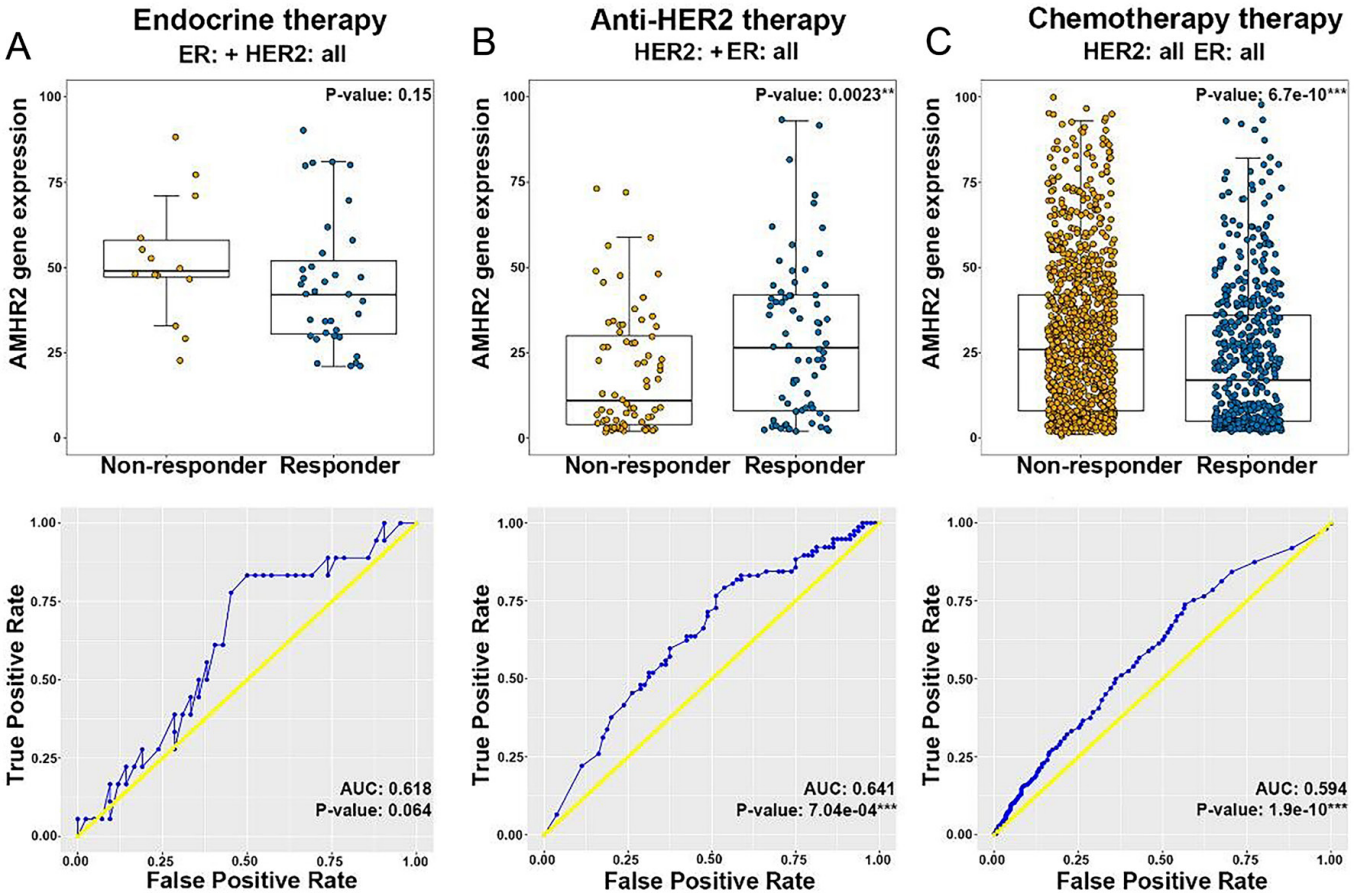
The AMHR2 gene expression in breast cancer patients with the different therapeutic responses for three kinds of treatments is different. (A) endocrine therapy; (B) anti-HER2 therapy; (C) chemotherapy. *P < 0.05, **P < 0.01, and ***P < 0.001. A full colour version of this figure can be found at https://doi.org/10.1530/ERC-23-0060.

According to the expression status of ER and HER2 in breast cancer patients and the corresponding treatment (endocrine therapy, anti-HER2 therapy, and chemotherapy), we use the receiver operating characteristics (ROC) plotter (http://www.rocplot.org/) to divide the breast cancer population into therapy responding group and non-responding group based on pathological complete response. Gene expression levels between responder and non-responder are compared. Area under curve (AUC) is analyzed and manufactured by ROC to explore whether AMHR2 can be used as a potential clinical biological marker to effectively predict therapeutic response. For ER+ breast cancer patients who have received endocrine therapy, the AMHR2 gene expression levels of the responding group and the non-responding group are similar, and the ROC *P*-value and Mann–Whitney test *P*-value both are over 0.05 (Fig. 6A). For HER2-positive patients who have received targeted therapy, AUC = 0.641, ROC *P*-value < 0.001, the responding group has higher AMHR2 gene expression (Mann–Whitney test *P*-value < 0.01, Fig. 6B) (Ben-Aharon *et al.* 2015, Morarji *et al.* 2017, Silva *et al.* 2019). In contrast, for patients who have received chemotherapy regardless of the phenotype of ER and HER2, AUC = 0.594, ROC *P*-value < 0.001, the non-responding group has higher AMHR2 gene expression (Mann–Whitney test *P*-value < 0.001, Fig. 6C). We speculate that the expression of ER and AMHR2 on the surface of breast cancer cells plays a positive role in promoting apoptosis when applying anti-HER2 therapy, and the AMHR2 on the surface of breast cancer cells may be related to chemotherapeutic drug resistance.

### Relationship between AMH/AMHRII and TP53 mutation and prognosis of breast cancer

**Figure 7:**
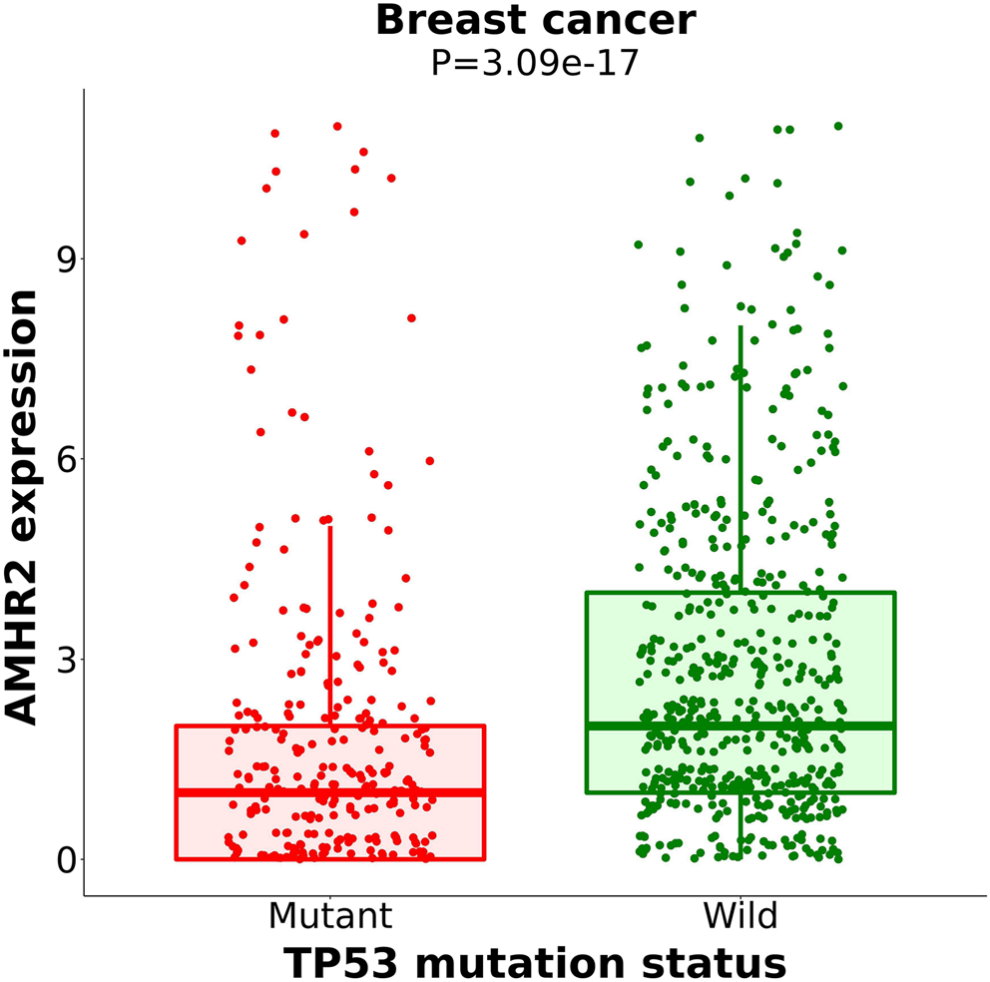
Patients suffering from breast cancer with different TP53 mutation statuses have different expressions of the AMHRII gene. Patients with a mutant TP53 gene had lower expression of the AMHRII gene than patients with a wildtype TP53 gene (*P* < 0.001).

TP53 has been recognized as a tumor suppressor gene, and its mutation can promote the occurrence and development of carcinoma (Soussi & Wiman 2015). Carriers of the TP53 mutant gene have a worse prognosis (Wang & Sun 2017). Studies have shown that TP53 mutations are independent markers of poor prognosis in breast cancer and several other cancers (Petitjean *et al.* 2007). The TP53 gene is altered in approximately 20–40% of breast cancers (Børresen-Dale 2003). We applied a cancer biomarker/target discovery tool (Nagy & Győrffy 2021) (http://www.mutarget.com/), which can identify mutations resulting in expression changes in the input gene, to explore whether mutations in other known genes alter the expression of AMHRII. It was found that patients suffering from breast cancer with a mutant TP53 gene had lower expression of the AMHRII gene (Fig. 7), which indirectly suggests that AMHRII may be a link in the downstream pathway after TP53 mutation. Mutant TP53 gene changes the expression of many genes through transcription factors, one of which may be to inhibit the expression of AMHRII gene in breast cancer cells to accelerate the growth and proliferation of breast cancer cells.

## Future avenues and perspectives

There is currently no gold-standard method for measuring AMH levels. This calls for efforts to develop a uniform method across countries for research and clinical application. In addition, AMH exhibits high variability among individuals due to the high variability in the number of antral follicles even within a group of subjects of the same age (Dewailly *et al.* 2014). AMH varies with ethnicity, lifestyle, and other factors. Although most studies mention that AMH does not fluctuate with the menstrual cycle, this view has been challenged with more accurate detection methods.

The interaction between AMH and other hormones involved in the hypothalamic–pituitary–ovarian axis needs to be explored. Although the expression of AMHRII in normal breast tissues and breast cancer cells has been reported, the mechanism of interaction between estrogen and AMHRII in breast cancer cells is not well understood. Additionally, further elucidation of the specific mechanism by which AMH inhibits the growth of breast cancer cells deserves more research.

The jury is still out on whether AMHRII can be incorporated into the molecular typing and diagnosis of breast cancer and be used to guide systemic treatment and individual prognosis counseling. It is also worth waiting to see whether AMH, AMH analogs, or AMHRII agonists can be used in the future as new drugs for breast cancer treatment without disrupting ovarian function (Salhi *et al.* 2004). In addition, AMH may be used as a delivery system for more toxic drugs to receptor-positive breast tumor cells, limiting exposure to nontarget tissues (MacLaughlin & Donahoe 2010). Covalently attaching cytotoxic agents to AMH with protease-sensitive linkages would allow the drug to be internalized along with AMH after receptor binding, and normally present cytosolic enzymes would cleave the drug from AMH, allowing it to function as usual (MacLaughlin & Donahoe 2010). We look forward to the development of AMH, its analog, AMHRII agonist, or anti-AMHRII antibodies, for the treatment of breast cancer and maintenance of ovarian function following antitumor systemic therapy.

## Conclusion

AMH specifically binds to AMHRII to activate downstream pathways and regulate gene transcription. Since AMHRII is expressed in breast cancer cells and triggers apoptosis, AMH/AMHRII will arouse innovation in the occurrence, typing, and targeted therapy of breast cancer (Table 1). The AMH level is a potent predictor of ovarian function after chemotherapy in premenopausal breast cancer patients older than 35 years, either for ovarian function injury or ovarian function recovery. Moreover, AMH may play an important role in the occurrence, treatment and prognosis of breast cancer, which needs further research.

**Table 1:** The roles of AMH in breast cancer.

	AMH/AMHRII in breast cancer
Occurrence	Laboratory studies: high concentrations of AMH can trigger cell apoptosis
	Epidemiological studies: no agreement.
	Result from database: higher AMHR2 gene expression indicates higher RFS.
Molecular typing	AMHRII may participate in molecular typing of breast cancer.
New remedy	Targeted AMHRII: synthetic AMH, AMH analog, anti-AMHRII antibody, AMHRII agonist, AMH as a delivery system with more toxic drugs.
Chemotherapy	AMH can reverse ovarian damage caused by chemotherapy.
	Combination of AMH and cytotoxic agents may be ideal for AMHRII-specific antineoplastic therapy.
Endocrine therapy	The reduction of estrogen levels caused by endocrine therapy decreases the inhibition of AMHRII expression on the surface of breast cancer cells.
	GnRHa suppresses mTOR pathway and activates autophagy which relieved ERS induced by CTX to increase the secretion of AMH.

AMH, anti-Müllerian hormone; AMHRII, anti-Müllerian hormone receptor II; CTX, cyclophosphamide; ERS, endoplasmic reticulum stress; GnRHa, gonadotropin-releasing hormone analogues; mTOR, mammalian target of rapamycin; RFS, relapse-free survival.

## Declaration of interest

All authors declare that they have no conflicts of interest.

## Funding

This project was funded by the Science and Technology Commission of Shanghai Municipality http://dx.doi.org/10.13039/501100003399 medical program (grant numbers 15411966500).

## Author contribution statement

All authors contributed to the manuscript and approved the final version. Chen X, Liu S, and Peng X contributed equally.
